# Predicting axillary residual disease after neoadjuvant therapy in breast cancer using baseline MRI and ultrasound

**DOI:** 10.1007/s00330-025-11408-4

**Published:** 2025-02-08

**Authors:** Caroline Malhaire, Ozgun Umay, Vincent Cockenpot, Fatine Selhane, Toulsie Ramtohul, Fabien Reyal, Jean-Yves Pierga, Emanuella Romano, Anne Vincent-Salomon, Youlia Kirova, Enora Laas, Hervé J. Brisse, Frédérique Frouin

**Affiliations:** 1 Institut Curie, Department of Medical Imaging, PSL Research University, 26 rue d’Ulm, 75005 Paris, France; 2 Institut Curie, LITO Laboratory, INSERM U1288, Paris-Saclay University, 91401 Orsay, France; 3 Institut Curie, Department of Pathology, 26 rue d’Ulm, 75005 Paris, France; 4 Gustave Roussy, Department of Imaging, 94800 Villejuif, France; 5 Institut Curie, Surgical Oncology Department, 26 rue d’Ulm, 75005 Paris, France; 6 Institut Curie, Medical Oncology, 26 rue d’Ulm, 75005 Paris, France; 7 Institut Curie, Department of Immunology, INSERM U932, PSL Research University, 75005 Paris, France; 8 Institut Curie, Department of Radiotherapy, University Versailles St Quentin, 26 rue d’Ulm, 75005 Paris, France

**Keywords:** Breast neoplasms, Tumour burden, Lymph nodes, Neoadjuvant therapy, Models, Statistical

## Abstract

**Objectives:**

To predict axillary node residual disease in women treated for node-positive breast cancer (BC) by neoadjuvant therapy (NAT), using breast BI-RADS MRI features and axillary ultrasound at baseline.

**Material and methods:**

In this single-center, retrospective study, women with node-positive BC who underwent NAT between 2016 and 2021 were included. Pre-treatment axillary US and breast MRIs were evaluated using the BI-RADS lexicon and T2 features, including Breast Edema Score. Univariate and multivariate logistic regression analyses were conducted for the prediction of axillary residual disease (ARD). A multivariable model based on logistic regression was trained and evaluated on randomly split train and test sets (7:3 ratio).

**Results:**

Out of the 141 women, 41% had post-NAT ARD. Axillary metastasis was independently associated with luminal subtype (odds ratio (OR), 25.5; *p* < 0.001), anterior tumor location (OR, 14.1; *p* = 0.008), and cortical thickening ≥ 7 mm (OR, 6.09; *p* = 0.002). Intratumoral T2 high signal intensity was protective (OR, 0.16; *p* = 0.006), while Ki67 had a marginal association (*p* = 0.064). In the training and test sets, the model, which is available online, achieved AUCs of 0.860 (95% CI: 0.783–0.936) and 0.843 (95% CI: 0.714–0.971), respectively. Anterior depth location and cortical thickening greater than 7 mm were also independently associated with post-NAT axillary burden.

**Conclusion:**

Adjusting for BC subtype and KI-67 index, the anterior third location of BC, a cortical thickness greater than 7 mm, and the absence of intratumoral T2 hyperintensity is predictive of ARD after NAT.

**Key Points:**

***Question*** *What baseline imaging-based predictive models can identify patients at risk of persistent nodal disease after neoadjuvant therapy?*

***Findings*** *Baseline US cortical thickness superior to 7 mm, anterior tumor location, and absence of an intratumoral high signal on T2-weighted MRI predict residual axillary disease.*

***Clinical relevance*** *Our predictive model, available online at:* litoic.shinyapps.io/LNPred_Apps, *including breast cancer subtype, Ki-67 index level, breast cancer location, intratumoral signal intensity on T2WI, and initial lymph node thickness, could guide post-NAT axillary management.*

**Graphical Abstract:**

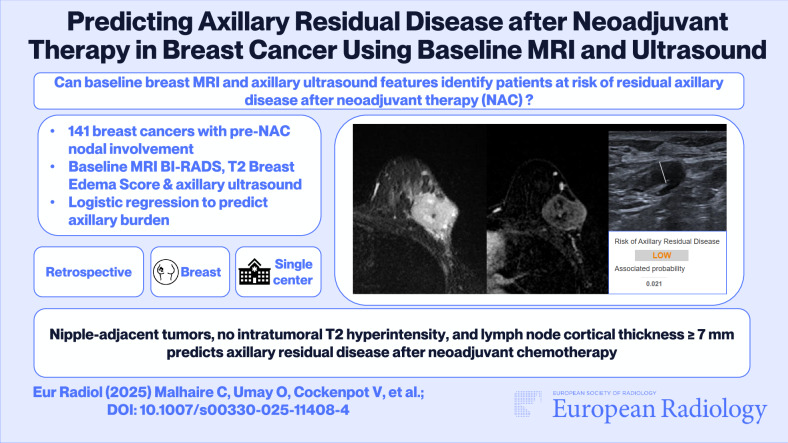

## Introduction

Neoadjuvant therapy (NAT) is increasingly used in breast cancer (BC) treatment. Achievement of pathologic complete response (pCR), defined as a complete tumor response in both breast and axilla [1], is associated with improved survival, mainly in triple-negative (TN) and HER2-positive BC. Targeted axillary dissection and optimized sentinel lymph node biopsy (SLNB) with dual-tracer mapping and removal of at least three lymph nodes (LN) are emerging as alternatives to axillary lymph node dissection (ALND) for patients who convert from node-positive to node-negative BC after NAT [2, 3]. However, concerns over residual axillary disease remain critical, as unresected metastatic nodes may compromise oncological outcomes. European guidelines currently limit axillary de-escalation to patients with cN1 pre-treatment stages, underscoring the need for reliable predictive models to inform these surgical strategies [4].

Imaging, particularly MRI, has been extensively explored for its ability to predict axillary response to NAT. However, its performance has shown limitations, with high false negative rates in post-NAT axillary assessments [5, 6]. Baseline imaging, by assessing the whole tumor, optimizes MRI’s ability to evaluate tumor characteristics. Post-NAT evaluations, in contrast, are limited by small residual foci and treatment-related changes, while baseline imaging supports tailored treatment planning. Recent advances, such as radiomics-based analysis of multiparametric MRI, have demonstrated the potential to improve predictive accuracy. A recent study highlighted the promise of radiomics in predicting axillary positive-node response to NAT [7]. Nevertheless, radiomics-based methods require image segmentation and processing, limiting their applicability in clinical workflows.

To address these challenges, our study aimed to develop a predictive model for axillary residual disease (ARD) using baseline breast MRI and axillary ultrasound, which do not require segmentation and can be readily applied. Additionally, we investigated how initial imaging characteristics correlate with post-NAT axillary burden using the Residual Cancer Burden (RCB) system, a standardized tool to quantify residual disease and a predictor of long-term outcomes [8].

## Materials and methods

### Study design and study population

This retrospective, single-center study performed in accordance with the Declaration of Helsinki was approved by our institutional review board, which waived the requirement for informed consent. We included BC patients treated with NAT between 2016 and 2021 who had nodal involvement, either clinically apparent ( ≥ cN1) or confirmed by cytology or histology, which were performed in cases of cortical thickening greater than 3 mm on ultrasound. Patients were not included if previously treated for ipsilateral BC, if they were pregnant or breastfeeding, or had breast implant(s).

Biological and MRI data from 75 patients were included in a previous study [9].

### Imaging protocols

At baseline axillary ultrasound, the LN maximum cortical thickness was measured perpendicularly to the LN long axis [10], or by measuring the short axis of the LN in the absence of a visible fatty hilum.

All MRI scans were performed in the prone position with a dedicated breast coil. The pre-treatment MRI scans of 113 patients were performed using a 1.5 T magnet, MAGNETOM Aera (Siemens), or a 1.5 T magnet Optima MR450w (General Electric Healthcare), as previously reported [9], with parameters in Supplemental Table S1. The MRI performed outside our institution in various centers for 28 patients were reviewed to control image quality and included at least axial T2WI with fat suppression and T1 fat-suppressed dynamic contrast-enhanced sequences and subtracted images in compliance with American College of Radiology recommendations with a slice thickness ≤ 3 mm and an in-plane resolution < 1 mm [11].

### Image analysis

MRI was reviewed in consensus by three radiologists (C.M., O.U., F.S.) blinded to the pathology results. MRI features were described using the BI-RADS lexicon [12].

Kinetic analysis was performed with time-intensity curves performed by placing a region-of-interest on the most enhancing area within the index mass, defined as the largest mass in cases of multiple lesions. Multifocality was defined as the presence of additional malignant sites within the ipsilateral breast, confirmed by biopsy as part of the initial staging workup. A non-mass enhancement (NME) associated with the index tumor was recorded when present. MRI sizes of the index mass and the maximal extension of multifocal lesions and/or NME were measured along the longest axis in the axial, coronal, or sagittal plane on the first subtracted axial images of dynamic contrast-enhanced MRI sequences. Tumor depth within the breast was recorded as indicated by the BI-RADS lexicon: anterior third, middle third, or posterior third of the breast.

Intratumoral high T2 signal intensity was defined as previously published as intensity equal to or greater than that of water or vessels [13]. Peritumoral, prepectoral, and subcutaneous edema were analyzed on T2WI to classify breast edema into four groups: BES1 (no edema), BES2 (peritumoral edema), BES3 (prepectoral edema), and BES4 (subcutaneous edema) [14].

### Clinical data and biological parameters

Clinical variables collected from medical records were age and clinical stage according to the TNM staging system. Neoadjuvant chemotherapy involved four cycles of anthracycline and cyclophosphamide, followed by paclitaxel administered weekly or docetaxel every three weeks. HER2-positive BC patients also received trastuzumab with chemotherapy.

Pathological variables collected from pre-treatment biopsies were histological type, grade, Ki-67 index, and Tumor-Infiltrating Lymphocytes (TILs) [15]. Hormonal receptor (HR) positivity was defined by a positive staining > 10%. HER2-positive BC was defined by overexpression via immunohistochemistry or fluorescence in situ hybridization. Luminal BC was identified as HR-positive/HER2-negative, TN BC as HR-negative/HER2-negative, and any HER2-positive BC, regardless of HR status, was defined as HER2-positive. Histological tumor response was assessed on post-NAT surgical specimens according to the RCB using the online calculator https://www3.mdanderson.org/app/medcalc/index.cfm?pagename=jsconvert3. A pathologic complete response (RCB0), according to the RCB, is defined by the absence of residual invasive lesions in the breast and axilla. RCB-I represents minimal residual disease, RCB-II represents moderate disease, and RCB-III has extensive residual disease. We recorded the number of axillary LN containing metastatic carcinoma, and the diameter of the largest metastasis in an axillary LN reported by pathologists to compute the RCB [16].

### Statistical analysis

Statistical analyses were performed using R software (version R-4.2.1). All tests were two-sided, and *p*-values < 0.05 were deemed significant. Continuous variables were compared using the Wilcoxon rank sum test, while categorical variables were compared using Pearson’s Chi-squared test or Fisher’s exact test, as appropriate. Baseline axillary ultrasound cortical thickening was dichotomized at a threshold of 7 mm, determined as the threshold with the lowest *p*-value in univariate analyses of cutoffs between 3 and 8 mm.

The association between imaging and clinical factors with residual axillary disease (ARD) was assessed using logistic regression. The study cohort was split into a 7:3 ratio for training and testing. Univariate logistic regression was initially performed on the training set. Variables with *p*-values < 0.1 were included in the multivariate model, which was finalized using backward stepwise selection.

The probability *p* of the event of interest (residual tumor burden) was defined by:

$$p= 	 \exp \left(z\right)/\left(1+\exp \left(z\right)\right){with}z= {a}_{0}+{a}_{1}.{TN}\\ 	 +{a}_{2}.{LUM}+{a}_{3}.{hKi}67 {+a}_{4}.{hT}2+{a}_{5}.{MID}\\ 	 +{a}_{6}.{ANT}+{a}_{7}.{hLNUS}$$

the coefficients ${a}_{{i}}$ and binary variables are being detailed in Table 1.

**Table 1:** Coefficients and definitions of binary variables used in the logistic regression model for predicting residual axillary disease

Coefficients	Description of binary variables
${a}_{0}=\,$−1.116	
${a}_{1}=\,$0.476	${TN}$ = 1 if the subtype of the breast cancer is Triple-Negative, 0 otherwise
${a}_{2}=\,$3.240	${LUM}$ = 1 if the subtype of the breast cancer is Luminal, 0 otherwise
${a}_{3}=\,$-1.401	${hKi}67$ = 1 if Ki-67 is greater than 25%, 0 otherwise
${a}_{4}=\,$-1.803	${hT}2$ = 1 if there is a high signal intensity inside the tumor on the T2-weighted image, 0 otherwise
${a}_{5}=\,$0.320	${MID}$ = 1 if the tumor is in the middle third, 0 otherwise
${a}_{6}=\,$2.643	${ANT}$ = 1 if the tumor is in the anterior third, 0 otherwise
${a}_{7}=\,$1.806	${hLNUS}$ = 1 if the size of the largest lymph node cortex on the ultrasound image is superior or equal to 7 mm, 0 otherwise

A conventional threshold of 0.5 is used to define positive and negative patients according to the value of the probability *p* of residual burden

Receiver operating characteristics (ROC) curves assessed model performance; Area under the roc curve (AUC) was calculated. optimal thresholds were derived using the youden index. Model AUC was tested against a null hypothesis of 0.5 using a *Z*-score. The added value of imaging features was evaluated using a likelihood ratio test by comparing multivariable model log-likelihoods via a chi-square test.

Calibration was evaluated using calibration curves, the Hosmer-Lemeshow test, and the Brier score in both datasets. Decision curve analysis assessed the clinical utility of the model. An HTML-based user interface was created to provide access to the predictive model via an online calculator (https://litoic.shinyapps.io/LNPred_Apps).

Ordinal logistic regression was used to analyze the association between imaging predictors and post-NAT axillary burden, categorized into three groups: no affected LDs, 1–3 affected LDs, and ≥ 4 affected LDs, testing whether baseline imaging could predict axillary involvement.

## Results

### Patient characteristics

We identified 148 patients eligible for inclusion in the study. Seven patients were excluded because of MRI technical failure (*n* = 2), missing data (*n* = 3), or if the MRI was performed after the first chemotherapy cycle (*n* = 2). Our study finally included 141 patients (mean age, 47 years; range, 39–55 years). After NAT completion, 84 patients (60%) achieved axillary complete response.

Clinical and pathological characteristics are presented according to axillary response in Table 2. Response rates differed significantly according to BC subtype (*p* < 0.001): the majority of luminal BC (9/38, 76%) did not achieve axillary response, whereas TN and HER2-positive BC were less likely to show ARD. High Ki-67 index and high TILs were significantly associated with axillary complete response. Patients with ARD composed almost the entirety of the RCB-III group and a major part of the RCB-II group. Four patients underwent completion ALND after nodal metastases were identified by SLNB. ALND yielded two additional metastatic LDs in one patient but was negative for the other three.

**Table 2:** Association between clinical and pathological characteristics and nodal response after neoadjuvant therapy

Characteristic	Axillary complete response, *N* = 84	Axillary residual disease, *N* = 57	*p*-value^a^
Age			0.149
< 40 y	27 (32%)	12 (21%)	
> 40 y	57 (68%)	45 (79%)	
T Stage			0.76°
0	16 (19%)	7 (12%)	
I	49 (58%)	33 (58%)	
II	14 (17%)	12 (21%)	
III	4 (5%)	4 (7%)	
IV	1 (1%)	1 (2%)	
N Stage			0.67°
0	19 (23%)	10 (18%)	
I	63 (75%)	45 (79%)	
II	2 (2%)	2 (3%)	
Tumor type			0.133°
Ductal NOS	81 (96%)	51 (89%)	
Lobular	0 (0%)	1 (2%)	
Mixt	0 (0%)	2 (4%)	
Other	3 (4%)	3 (5%)	
ER Status			**< 0.001**
Negative	58 (69%)	21 (37%)	
Positive	26 (31%)	36 (63%)	
PR Status			**< 0.001**
Negative	65 (77%)	28 (49%)	
Positive	19 (23%)	29 (51%)	
BC Subtypes			**< 0.001**
HER2+	31 (37%)	14 (25%)	
TNBC	44 (52%)	14 (25%)	
Luminal	9 (11%)	29 (51%)	
Grade			0.15
1/2	26 (31%)	24 (43%)	
3	58 (69%)	32 (57%)	
(Missing)	0	1	
Ki-67			**< 0.001**
≤ 25%	6 (7.1%)	16 (28%)	
> 25%	78 (93%)	41 (72%)	
TILs			**0.027**
≤ 30%	55 (65%)	47 (82%)	
> 30%	29 (35%)	10 (18%)	
RCB Class			**< 0.001**
pCR	52 (62%)	0 (0%)	
RCB-I	15 (18%)	4 (7%)	
RCB-II	16 (19%)	28 (49%)	
RCB-III	1 (1%)	25 (44%)	
*n* (%)			

The bold indicates significant results, *p*-value inferior to 0.05

^a^ Pearson’s Chi-squared test was used unless subgroup sizes were too small; in these cases, Fisher’s exact test was applied. Parameters tested using Fisher’s exact test are marked with an circle (°)

### Association of imaging features with nodal response

Table 3 summarizes the association of imaging features with ARD in the study population.

**Table 3:** Association between imaging characteristics of the study population and nodal response after neoadjuvant therapy

Characteristic	Axillary complete response, *N* = 84	Axillary residual disease, *N* = 57	*p*-value^a^
US cortical thickness			**< 0.001**
< 7 mm	63 (75%)	26 (46%)	
≥ 7 mm	21 (25%)	31 (54%)	
Depth localization			**0.012**
Posterior third	45 (54%)	18 (32%)	
Middle third	34 (40%)	29 (51%)	
Anterior third	5 (6%)	10 (18%)	
Breast composition			0.181°
A	4 (5%)	3 (5.3%)	
B	38 (45%)	32 (56%)	
C	23 (27%)	17 (30%)	
D	19 (23%)	5 (8.8%)	
Background parenchymal enhancement			0.749
Minimal/Mild	69 (82%)	48 (84%)	
Moderate/Marked	15 (18%)	9 (16%)	
Margins			**0.028**
Circumscribed/Irregular	54 (64%)	26 (46%)	
Spiculated	30 (36%)	31 (54%)	
Shape			**0.021**
Irregular	58 (69%)	49 (86%)	
Oval/Round	26 (31%)	8 (14%)	
Intratumoral high SI on T2			0.135
Present	29 (35%)	13 (23%)	
Absent	55 (65%)	44 (77%)	
Peritumoral edema			0.08
Present	67 (80%)	38 (67%)	
Absent	17 (20%)	19 (33%)	
Prepectoral edema			0.518
Present	34 (40%)	20 (35%)	
Absent	50 (60%)	37 (65%)	
Subcutaneous edema			0.271
Present	13 (15%)	13 (23%)	
Absent	71 (85%)	44 (77%)	
BES			0.2
1	16 (19%)	17 (30%)	
2	31 (37%)	16 (28%)	
3	24 (29%)	11 (19%)	
4	13 (15%)	13 (23%)	
Multifocality			0.43
Present	27 (32%)	22 (39%)	
Absent	57 (68%)	35 (61%)	
Associated non-mass enhancement			**0.003**
Absent	68 (81%)	33 (58%)	
Present	16 (19%)	24 (42%)	
Internal enhancement type			0.969
Homogeneous	26 (31%)	17 (30%)	
Heterogeneous	38 (45%)	27 (47%)	
Rim Enhancement	20 (24%)	13 (23%)	
Delayed phase enhancement			0.336°
Persistent	3 (4%)	4 (7%)	
Plateau	13 (15%)	13 (23%)	
Wash-out	68 (81%)	40 (70%)	
Index Lesion MR Size	26 (21, 41)	28 (22, 38)	0.762^
Maximal MR Size	33 (24, 47)	39 (25, 66)	**0.049^**
*n* (%); Median (IQR)			

The bold indicates significant results, *p*-value inferior to 0.05

^a^ Pearson’s Chi-squared test was used unless subgroup sizes were too small; in these cases, Fisher’s exact test was applied. Parameters tested using Fisher’s exact test are marked with (°). Continuous variables, marked with (^), were compared using the Wilcoxon rank sum test

All tumors included presented mass-like enhancements on MRI with a median index tumor size of 27 mm (IQR: 21–39 mm). Among patients who achieved a complete response, 75% had a baseline cortical thickness < 7 mm (*p* < 0.001). Tumors located in the anterior third of the breast (*p* = 0.012), with an irregular shape (*p* = 0.021), spiculated margins (*p* = 0.028), a non-mass enhancement (*p* = 0.003), and a larger maximal MR extension (39 mm vs. 33 mm, *p* = 0.049) were associated with ARD.

### Residual axillary disease prediction

**Fig. 1:**
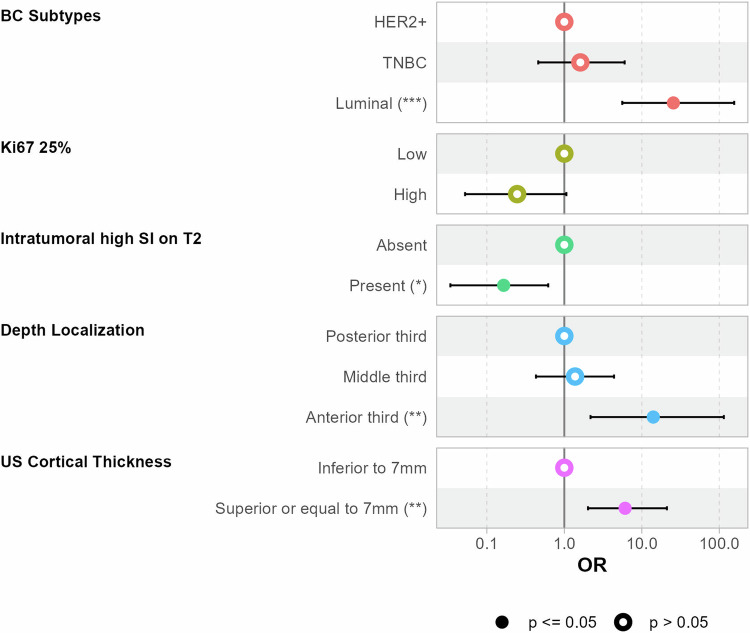
Forest plot for the multivariable logistic regression full model in the training and test sets

Characteristics of the entire population and a comparison between training and test sets are given in Table S2. Multivariate logistic regression for predicting ARD in the training set (Table 4) indicated differential odds among BC subtypes. The luminal subtype was associated with significantly increased odds of ARD compared to HER2-positive BC (odds ratio (OR), 25.5; 95% CI, 5.60–156; *p* < 0.001). An intratumoral high signal intensity on T2WI was inversely associated with ARD (OR 0.16; 95% CI (0.03–0.62); *p* = 0.006). Cortical thickness greater than 7 mm was associated with higher odds of ARD (OR, 6.09; 95% CI, 2.02–21.1, *p* = 0.002). Lesion depth in the breast also affected the odds, with the anterior third showing significantly increased odds of ARD compared to the posterior third (reference level) (OR, 14.1; 95% CI, 2.18–115; *p* = 0.008), while the middle third showed no significant difference (Fig. 1). Adding imaging characteristics to a model including the Ki-67 index and BC subtype significantly enhanced model performance (*p* < 0.001).

**Table 4:** Multivariate logistic regression analysis for prediction of residual axillary disease in the training set

	Multivariate logistic regression			
Characteristic	OR	95% CI	*p*-value	p*
BC Subtypes			**< 0.001**	
HER2+	—	—		
TNBC	1.61	0.46, 6.01		0.5
Luminal	25.5	5.60, 156		**< 0.001**
Ki-67			0.06	
≤ 25%	—	—		
> 25%	0.25	0.05, 1.06		
US cortical thickness			**0.002**	
< 7 mm	—	—		
≥ 7 mm	6.09	2.02, 21.1		
Depth localization			**0.02**	
Posterior third	—	—		
Middle third	1.38	0.43, 4.38		0.6
Anterior third	14.1	2.18, 115		**0.008**
Intratumoral high SI on T2			**0.006**	
Absent	—	—		
Present	0.16	0.03, 0.62		

The bold indicates significant results, *p*-value inferior to 0.05

*OR* odds Ratio, *CI* confidence Interval

**Fig. 2:**
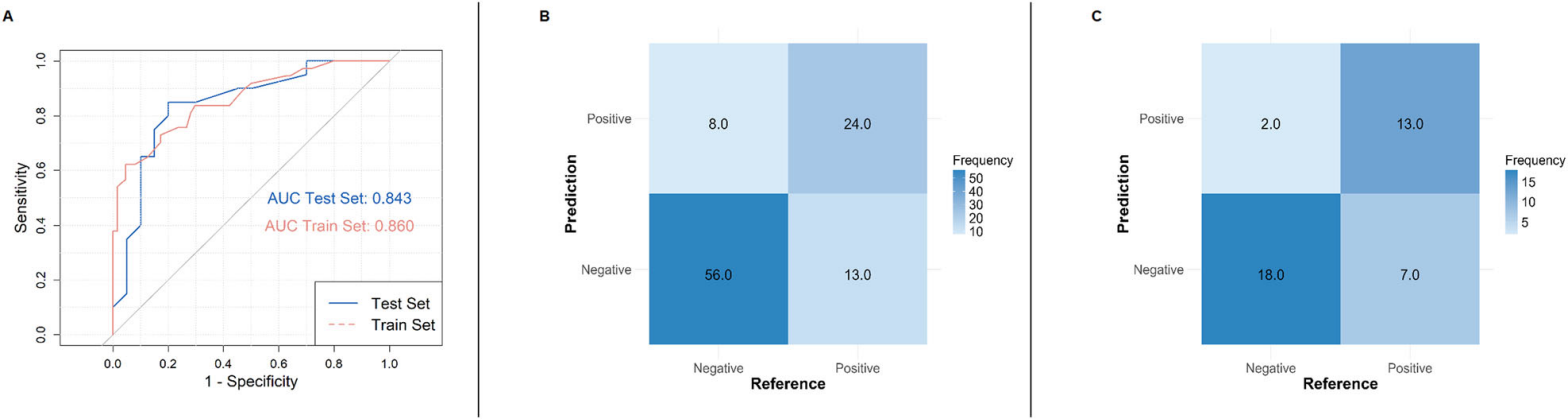
Performance of the full model for predicting lymph node residual disease. Receiver operating characteristic curves show lymph node status prediction by the model in the training set (red curve) and the test set (blue curve) (**A**). Confusion matrices show lymph node true status versus prediction. for the training set (**B**) and the test set (**C**). AUC, area under curve; ROC, receiver operating characteristic curve

In the training set, the full model displayed significant discriminative capacity, achieving an AUC of 0.860 (95% CI: 0.783–0.936, *p* < 0.001). The accuracy of predicting ARD reached 79% with 65% sensitivity, 87% specificity, 75% positive predictive value (PPV), and 81% negative predictive value (NPV). The full model in the test set had an AUC of 0.843 (95% CI: 0.714–0.971, *p* = 0.006) and showed 78% accuracy, 65% sensitivity, 90% specificity, 87% PPV, and 72% NPV. Figure 2 displays the ROC curves and confusion matrices for both datasets.

The full model demonstrated good calibration (*p* = 0.65 in training, *p* = 0.09 in testing) and Brier scores of 0.27 in training and 0.31 in testing. Calibration plots showed a satisfying correlation between observed and predicted ARD in both sets (Fig.  S1). Decision curve analysis indicated that the full model achieved greater net benefits than both the assume-all and assume-none approaches in both sets (Fig.  S2).

### Post-NAT axillary burden

Considering axillary metastasis burden following NAT, we showed that depth location and ultrasound cortical thickness were significantly associated with the post-NAT LN metastases number. Table 5 presents the results of a univariate and multivariate ordinal logistic regression analysis comparing the presence of 0, 1 to 3, and 4 or more LN across clinical and imaging characteristics. Anterior location in the breast and large cortex thickness were independent predictors of a higher post-NAC axillary stage. Intratumoral high SI on T2WI showed a non-significant trend towards association with LN count. In the univariate analysis, a larger MR size, an associated NME, spiculated margins, an irregular shape, and the absence of peritumoral edema were associated with an increased LN count.

**Table 5:** Association between imaging features and axillary residual burden as assessed by the RCB system and by the number of axillary lymph nodes affected after neoadjuvant chemotherapy, grouped into three categories: no affected lymph node, 1–3 involved lymph nodes, and more than 4 nodes

	Axillary metastastic number after NAT			Univariate ordinal logistic regression				Multivariate ordinal logistic regression			
Characteristic	0, *N* = 84	1 to 3 LN, *N* = 41	4 or more LN, *N* = 16	OR	95% CI	*p*-value	p*	OR	95% CI	*p*-value	p*
Ki-67						**< 0.001**				**< 0.001**	
≤ 25%	6 (7%)	10 (24%)	6 (38%)	—	—			—	—		
> 25%	78 (93%)	31 (76%)	10 (63%)	0.21	0.09, 0.51			0.17	0.06, 0.46		
BC subtypes						**< 0.001**				**< 0.001**	
HER2+	31 (37%)	14 (34%)	0 (0%)	—	—			—	—		
TNBC	44 (52%)	11 (27%)	3 (19%)	0.77	0.32, 1.84		0.6	1,57	0.58, 4.41		0.4
Luminal	9 (11%)	16 (39%)	13 (81%)	9.12	3.69, 24.0		**< 0.001**	19.3	6.68, 62.4		**< 0.001**
US cortical thickness						**< 0.001**				**< 0.001**	
< 7 mm	63 (75%)	23 (56%)	3 (19%)	—	—			—	—		
≥ 7 mm	21 (25%)	18 (44%)	13 (81%)	4,22	2.11, 8.62			6,94	3.01, 16.9		
Depth localization						**0.011**				** 0.009**	
Posterior third	45 (54%)	13 (32%)	5 (31%)	—	—			—	—		
Middle third	34 (40%)	22 (54%)	7 (44%)	2.03	0.99, 4.23		0.055	1,59	0.67, 3.85		0.3
Anterior third	5 (6.0%)	6 (15%)	4 (25%)	5.01	1.67, 15.3		**0.004**	9,7	2.59, 37.9		**< 0.001**
Breast composition						0.2					
A	4 (5%)	2 (4.9%)	1 (6%)	—	—						
B	38 (45%)	24 (59%)	8 (50%)	1.05	0.24, 5.52						
C	23 (27%)	11 (27%)	6 (38%)	1	0.21, 5.51						
D	19 (23%)	4 (9.8%)	1 (6%)	0.34	0.06, 2.11						
Background parenchymal enhancement						0.6					
Minimal	44 (52%)	22 (54%)	9 (56%)	—	—						
Mild	25 (30%)	15 (37%)	2 (13%)	0.87	0.41, 1.81						
Moderate	6 (7%)	3 (7%)	3 (19%)	1.69	0.49, 5.58						
Marked	9 (11%)	1 (2%)	2 (13%)	0.55	0.11, 2.03						
Margins						**0.01**					
Circumscribed/Irregular	54 (64%)	22 (54%)	4 (25%)	—	—						
Spiculated	30 (36%)	19 (46%)	12 (75%)	2.42	1.24, 4.76						
Shape						**0.018**					
Irregular	58 (69%)	34 (83%)	15 (94%)	—	—						
Oval / Round	26 (31%)	7 (17%)	1 (6%)	0.35	0.14, 0.80						
Intratumoral high SI on T2						0.3				0.06	
Absent	55 (65%)	35 (85%)	9 (56%)	—	—			—	—		
Present	29 (35%)	6 (15%)	7 (44%)	0.68	0.31, 1.44			0.4	0.15, 1.01		
Peritumoral edema						**0.049**					
Absent	17 (20%)	12 (29%)	7 (44%)	—	—						
Present	67 (80%)	29 (71%)	9 (56%)	0.48	0.23, 1.00						
Prepectoral edema						0.7					
Absent	50 (60%)	28 (68%)	9 (56%)	—	—						
Present	34 (40%)	13 (32%)	7 (44%)	0.86	0.43, 1.69						
Subcutaneous edema						0.086					
Absent	71 (85%)	35 (85%)	9 (56%)	—	—						
Present	13 (15%)	6 (15%)	7 (44%)	2.1	0.89, 4.89						
BES						0.08					
1	16 (19%)	12 (29%)	5 (31%)	—	—						
2	31 (37%)	14 (34%)	2 (13%)	0.46	0.19, 1.11						
3	24 (29%)	9 (22%)	2 (13%)	0.43	0.16, 1.10						
4	13 (15%)	6 (15%)	7 (44%)	1.21	0.44, 3.29						
Multifocality						0.5					
Present	27 (32%)	16 (39%)	6 (38%)	—	—						
Absent	57 (68%)	25 (61%)	10 (63%)	0.77	0.39, 1.54						
Associated non-mass enhancement						**0.004**					
Absent	68 (81%)	24 (59%)	9 (56%)	—	—						
Present	16 (19%)	17 (41%)	7 (44%)	2.85	1.40, 5.84						
Internal enhancement Type						> 0.9					
Homogeneous	26 (31%)	14 (34%)	3 (19%)	—	—						
Heterogeneous	38 (45%)	19 (46%)	8 (50%)	1.16	0.55, 2.52						
Rim Enhancement	20 (24%)	8 (20%)	5 (31%)	1.13	0.45, 2.80						
Delayed phase enhancement						0.4					
Persistent	3 (3.6%)	4 (9.8%)	0 (0%)	—	—						
Plateau	13 (15%)	9 (22%)	4 (25%)	1.05	0.24, 5.01						
Wash-out	68 (81%)	28 (68%)	12 (75%)	0.63	0.17, 2.69						
Index lesion MR size	26 (21, 41)	26 (21, 35)	36 (24, 51)	1.01	0.99, 1.04	0.4					
Maximal MR size	33 (24, 47)	36 (24, 59)	45 (31, 72)	1.02	1.00, 1.03	**0.008**					

The bold indicates significant results, *p*-value inferior to 0.05

*n* (%); Median (IQR)

Fisher’s exact test; Pearson’s Chi-squared test; Kruskal–Wallis rank sum test

*OR* odds Ratio, *CI* confidence Interval

**Fig. 3:**
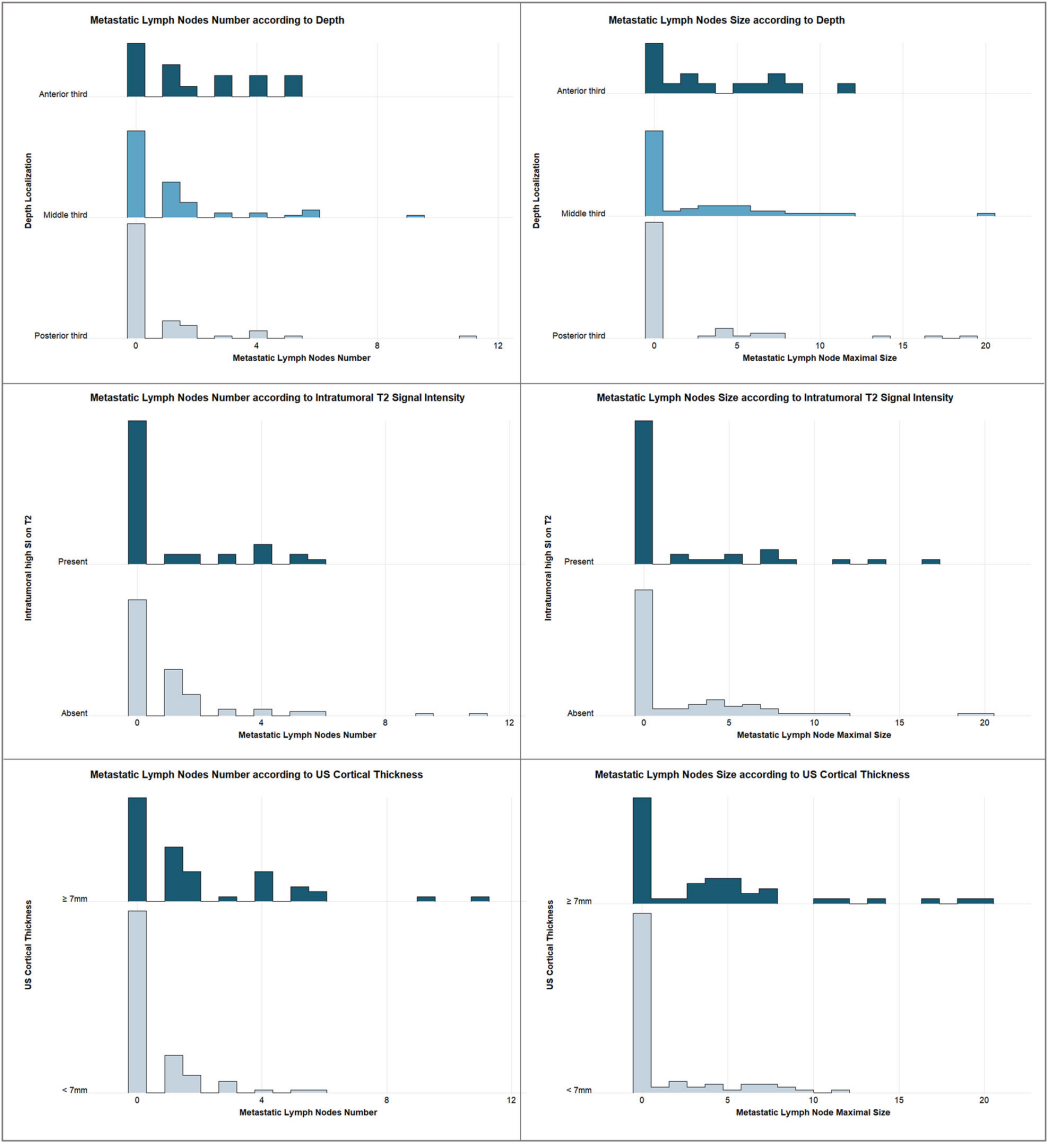
Post-neoadjuvant metastatic LN number and maximum size across significant imaging characteristics

The average diameter of the largest LN metastasis significantly increased from the posterior third (mean = 1.72 mm, SD = 4.06) to the anterior third (mean = 3.53 mm, SD = 3.76; *p* = 0.009). Patients with cortical thickness ≥ 7 mm had larger LN metastases (mean = 3.87 mm, SD = 5.17) than those with thickness < 7 mm (mean = 1.28 mm, SD = 2.62; *p* < 0.001). There was no significant difference in the largest LN metastasis diameter between cases with high T2 SI (mean = 2.21 mm, SD = 4.18) and without (mean = 2.27 mm, SD = 2.47; *p* = 0.544). Figure 3 depicts the number and maximum size of metastatic LN across all imaging characteristics.

### Breast cancer subtypes

Clinicopathological and imaging features associations with nodal response to NAT stratified by BC subtype are presented in Table S3.

In TNBC, the absence of NME was associated with axillary complete response, and the presence of peritumoral edema demonstrated a trend towards significance (*p* = 0.052).

**Fig. 4:**
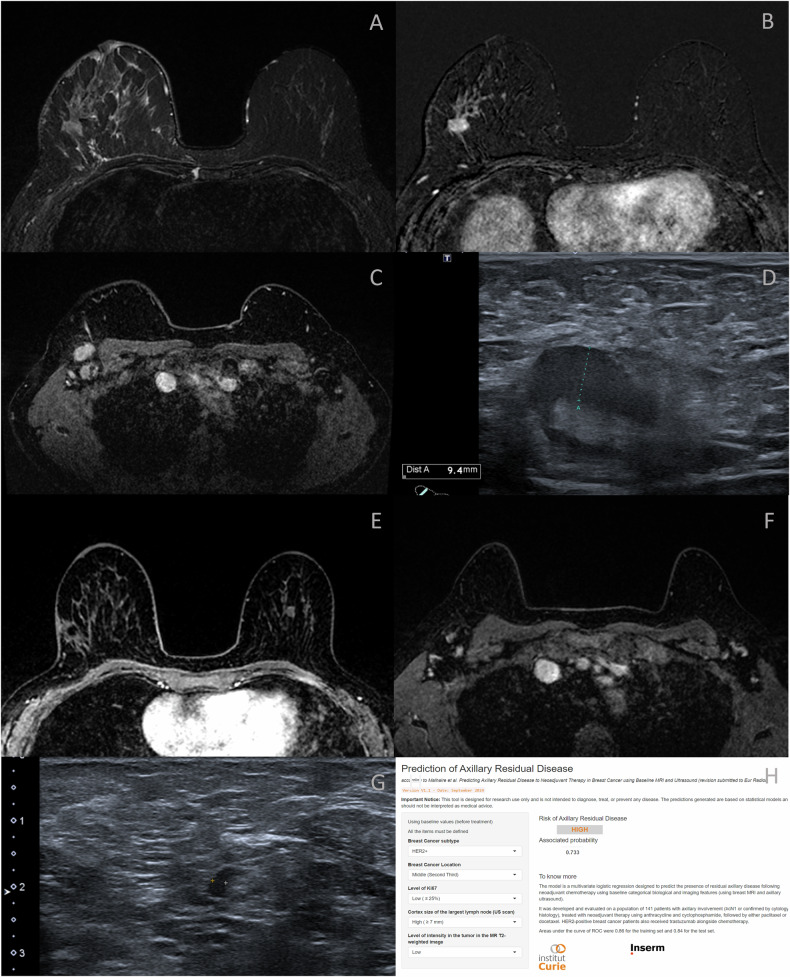
Breast MRI of a patient with HER2-positive breast carcinoma and Ki-67 25%. The axial T2-weighted image shows no central T2 hyperintensity inside the mass (**A**). On T1-weighted contrast-enhanced subtracted images, the tumor is located in the middle third of the breast, accompanied by a non-mass enhancement (NME) oriented toward the nipple (**B**). Contrast-enhanced T1-weighted MRI demonstrates large axillary adenopathies (**C**), with cortical thickening measuring up to 9 mm on ultrasound (**D**). Following NAT, dynamic contrast-enhanced T1-weighted sequences (**E**) show no residual enhancement around the intratumoral clip. Lymph node thickness has normalized on MRI (**F**) and ultrasound (**G**) with a 2.5 mm cortical thickness. However, postoperative histology confirmed 2 positive nodes out of 5, including an 11 mm macrometastasis, without breast residual disease, ypT0N1a. Residual Cancer Burden (RCB) was classified as RCB II. The axillary residual disease was accurately predicted by the model (**H**)

**Fig. 5:**
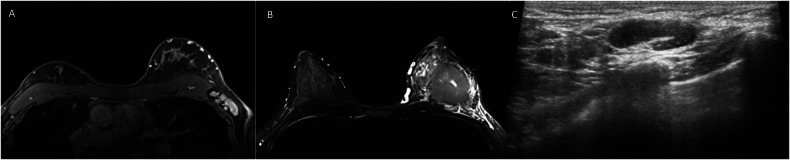
Pre-therapeutic imaging of a 37-year-old patient with luminal breast carcinoma (Ki-67 80%). The tumor is located in the middle third of the breast, presenting as a round mass with non-spiculated margins an intratumoral T2 hyperintensity, and a peritumoral edema on axial T2-weighted image (**A**). Contrast-enhanced T1-weighted sequences demonstrate multiple axillary metastases (**B**) with a maximum cortical thickness of 4.4 mm at baseline axillary ultrasound (**C**). After NAT, total mastectomy and ALND show complete breast and axillary response

**Fig. 6:**
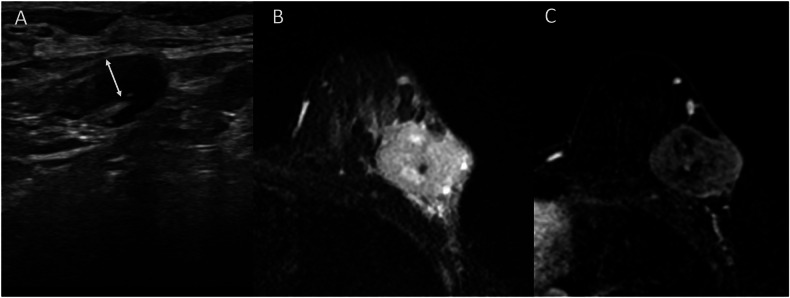
Triple-negative breast cancer in a 44-year-old patient, with a KI-67 equal to 90%. Baseline axillary ultrasound shows an adenopathy with a 4 mm cortical thickening (**A**). On MRI examination, the tumor in the posterior part of the breast includes high central signal intensity areas. It is surrounded by peritumoral edema on T2-weighted images (**B**), with a heterogeneous enhancement without non-mass enhancement on subtracted images from dynamic contrast-enhanced T1-weighted acquisitions (**C**). The patient achieved an axillary complete response after neoadjuvant chemotherapy, RCB0. RCB, residual cancer burden

In HER2-positive BC, a high Ki-67 index was significantly associated with nodal response after NAT, and a trend towards an association was noted for cortical thickening (*p* = 0.053). In luminal BC, internal enhancement patterns were the most notable imaging feature associated with ARD, with a trend toward statistical significance (*p* = 0.089). Illustrative examples are provided in Figs. 4–6.

## Discussion

Our findings demonstrate that BC patients presenting with anterior tumors, no intratumoral high signal intensity on T2WI, and baseline ultrasound LD cortical thickening greater than 7 mm exhibit a higher risk of residual axillary disease post-neoadjuvant therapy. A combined model that included these imaging features, along with the BC subtype and Ki-67 index, achieved a significantly higher AUC than a model incorporating only pathologic variables.

Post-NAT US and MRI axillary evaluations are of low predictive value, with false negative rates superior to that of SLNB, highlighting the risk of relying solely on post-NAT imaging to assess axillary response [13].

Prior studies, such as that by Kim et al [17] evaluated axillary response by integrating both baseline and post-NAT factors, including the change in tumor size observed on MRI, LN characteristics assessed by ultrasound. Their model incorporated a decrease in MRI BC size of less than 50%, ultrasound-measured post-NAT LN cortex greater than 3.5 mm, initial nodal clinical stage N2-N3, negative HER2 status, positive ER status, and a low Ki-67 index, achieving AUCs of 0.84 in the training set and 0.78 in the validation set. To our knowledge, our study is the first to assess the ability of baseline tumor characteristics, including BI-RADS features and breast T2WI descriptors, to predict ARD before the initiation of therapy. Developing predictive criteria at baseline is critical for enabling personalized treatment strategies, as it allows oncologists to tailor treatment strategies before initiating therapy.

Anterior location in the breast exhibited a high OR for ARD and was an independent predictor of axillary burden after NAT. This is consistent with previous reports of increased axillary invasion in tumors located close to the nipple, independent of patient age, biologic subtype, or size in the adjuvant setting [18].

Conflicting findings have been reported regarding the relationship between intratumoral high signal intensity areas on MRI and the response to NAT. High signal intensity on T2WI is associated with TNBC subtype, high tumor grade, and larger tumor size [19, 20]. Uematsu et al reported an association of high signal intensity on T2WI with poor response to NAT in a population of various BC subtypes [21]. These authors used response criteria not specifically addressing the nodal response. In a study including only TNBC from the non-specific subtype, this association was not confirmed, possibly due to a small study sample size and the exclusion of TN subtypes such as metaplastic TNBC, which are known to be associated with intratumoral necrosis and poor responders [13]. In a recent study with higher rates of pCR, more representative of response rates in modern therapeutic regimens, Abdelhafez et al showed no association between necrosis in TNBC and pCR or axillary staging before or after NAT [22].

In TNBC, we showed that the absence of NME was associated with axillary complete response, while the presence of peritumoral edema demonstrated a trend towards significance. NME surrounding malignant index mass is a feature previously described in association with low KI-67 index [23], whereas peritumoral edema has been reported in association with increased KI-67 index, high histological grade [24], as well as with recurrence-free survival [25, 26].

Our findings additionally demonstrate that axillary tumor burden after NAT, a key prognostic factor, is associated with tumor depth, baseline cortical thickening, and intratumoral signal intensity on T2WI. While the RCB index incorporates both breast and axillary response [6], the evidence suggests that ARD is the component that mostly contributes to its prognostic value [27, 28].

The limits of our study are its single-center retrospective design and its small test set. The semantic features included in the variables studied may lead to subjectivity. Nonetheless, they are based on the BI-RADS lexicon and breast edema descriptors, which have been developed and updated to enhance interreader reliability, making them highly transferable features in daily practice. Due to the size of the study population, our ability to perform subgroup analyses by BC subtypes was limited.

## Conclusion

Anterior tumor location, US cortical thickness superior to 7 mm, and a lack of intratumoral T2 high signal intensity are independent predictors of ARD after NAT. These features improve the clinical prediction model based on the BC subtype and KI-67 index. These features are also associated with post-NAT axillary burden. Non-mass enhancement is associated with ARD in TNBC, while the absence of peritumoral edema shows a trend towards this association, consistent with their link to low Ki-67 levels and less proliferative tumors. Larger studies are needed to validate our results across BC subtypes and help identify patients who are at risk of ARD to guide axillary de-escalation.

## Supplementary information

ELECTRONIC SUPPLEMENTARY MATERIAL

## Abbreviations

ALND: Axillary lymph node dissection
ARD: Axillary residual disease
AUC: Area under curve
BC: Breast cancer
BES: Breast Edema Score
CI: Confidence interval
HR: Hormonal receptor
LN: Lymph node
NAT: Neo-adjuvant therapy
NME: Non-mass enhancement
OR: Odds ratio
pCR: Pathologic complete response
RCB: Residual cancer burden
ROC: Receiver operating characteristic
SD: Standard deviation
SLNB: Sentinel lymph node biopsy
TILs: Tumor-infiltrating lymphocytes
TNBC: Triple-negative breast cancer
