# Supplementary material for: Predicting axillary residual disease after neoadjuvant therapy in breast cancer using baseline MRI and ultrasound
Source: Eur Radiol. 2025 Feb 8;35(8):4896–909. doi: 10.1007/s00330-025-11408-4 (PMC12226675; doi:10.1007/s00330-025-11408-4)
Supplement: Supplementary file 1 — ELECTRONIC SUPPLEMENTARY MATERIAL [file 330_2025_11408_MOESM1_ESM.pdf]

Predicting Axillary Residual Disease after Neoadjuvant Therapy in Breast Cancer Using Baseline MRI and Ultrasound

ELECTRONIC SUPPLEMENTARY MATERIAL

Table S1. Breast MRI acquisition parameters

Coil	Coil 1*, N=21		Coil 2**, N=52		Coil 3***, N=40	
	T2-weighted sequence	T1-weighted DCE sequence	T2-weighted sequence	T1-weighted DCE sequence	T2-weighted sequence	T1-weighted DCE sequence
TR (ms)	3310	5.2	6400	5.2	5544	6.81
TE (ms)	88	2.4	88	2.4	90	3.3
Slice thickness (mm)	3.5	0.9	3	0.9	3	1
Slice interval (mm)	4.2	0.9	3.6	0.9	3.3	1
Pixel spacing (mm)	0.7x0.7	0.91x0.91	0.7x0.7	0.91x0.91	0.68x0.68	0.82x0.82
Pixel bandwidth (Hz/pix)	315	355	375	355	558	434
Flip angle (°)	150	10	180	10	160	15
Field of View (mm^2)	360x360	380x342	360x360	380x380	350x350	420x420
Matrix	512x435	416x312	512x435	416x312	416x416	416x416

*Coil 1: 18-channel coil, 1.5 T magnet, MAGNETOM Aera (Siemens)
**Coil 2: 16-channel coil (Sentinelle; Philips Medical Systems), MAGNETOM Aera (Siemens)
***Coil 3: 8-channel coil, 1.5 T magnet, Optima MR450w (GE)
DCE: dynamic contrast-enhanced, TE: time to echo, TR: repetition time

Table S2. Study population, training, and test cohorts' characteristics.

Characteristic	Overall, N = 141	Train Set, N = 101	Test Set, N = 40	*p*-value
**Age**				0.419
≤ 40y	39 (28%)	26 (26%)	13 (33%)	
> 40y	102 (72%)	75 (74%)	27 (68%)	
**T Stage**				0.134
0	23 (16%)	21 (21%)	2 (5%)	
I	82 (58%)	55 (54%)	27 (68%)	
II	26 (18%)	18 (18%)	8 (20%)	
III	8 (6%)	5 (5%)	3 (7%)	
IV	2 (2%)	2 (2%)	0 (0%)	
**N Stage**				0.322
0	29 (21%)	19 (19%)	10 (25%)	
I	108 (77%)	80 (79%)	28 (70%)	
II	4 (2%)	2 (2.0%)	2 (5.0%)	
**Tumor type**				0.88
Ductal NOS	132 (94%)	93 (92%)	39 (98%)	
Lobular	1 (1%)	1 (1%)	0 (0%)	
Mixt	2 (1%)	2 (2%)	0 (0%)	
Other	6 (4%)	5 (5%)	1 (2%)	
**ER Status**				0.825
Negative	79 (56%)	56 (55%)	23 (58%)	
Positive	62 (44%)	45 (45%)	17 (43%)	
**PR Status**				0.586
Negative	93 (66%)	68 (67%)	25 (63%)	
Positive	48 (34%)	33 (33%)	15 (38%)	
**BC Subtypes**				0.333
HER2+	45 (32%)	35 (35%)	10 (25%)	
TNBC	58 (41%)	42 (42%)	16 (40%)	
Luminal	38 (27%)	24 (24%)	14 (35%)	
**Grade**				0.911
1/2	50 (36%)	36 (36%)	14 (35%)	

3	90 (64%)	64 (64%)	26 (65%)	
**Ki67**				0.696
≤ 25%	22 (16%)	15 (15%)	7 (18%)	
> 25%	119 (84%)	86 (85%)	33 (83%)	
**TILs**				**0.039**
≤ 30%	102 (72%)	78 (77%)	24 (60%)	
> 30 %	39 (28%)	23 (23%)	16 (40%)	
**Breast Surgery Type**				0.064
Partial Mastectomy	94 (67%)	72 (71%)	22 (55%)	
Total Mastectomy	47 (33%)	29 (29%)	18 (45%)	
**Axillary Surgery Type**				0.688
Axillary LA	106 (75%)	75 (74%)	31 (78%)	
Sentinel Node Biopsy	35 (25%)	26 (26%)	9 (23%)	
**RCB Class**				0.991
pCR	52 (37%)	38 (38%)	14 (35%)	
RCB-1	19 (13%)	13 (13%)	6 (15%)	
RCB-II	44 (31%)	32 (32%)	12 (30%)	
RCB-III	26 (18%)	18 (18%)	8 (20%)	
**Post NAC LN Status**				0.145
Axillary Complete Response	84 (60%)	64 (63%)	20 (50%)	
Axillary Residual Disease	57 (40%)	37 (37%)	20 (50%)	
n (%)				
Pearson's Chi-squared test; Fisher's exact test				

Table S3. Association of clinicopathological and imaging characteristics with nodal response to neoadjuvant therapy in breast cancer subtypes.

	**Luminal**			**HER2+**			**TNBC**		
**Characteristic**	**Axillary Complete Response, N = 9**	**Axillary Residual Disease, N = 29**	***p*-value**	**Axillary Complete Response, N = 31**	**Axillary Residual Disease, N = 14**	***p*-value**	**Axillary Complete Response, N = 44**	**Axillary Residual Disease, N = 14**	***p*-value**
**Ki67**			0.164			**0.049**			0.085
≤ 25%	0 (0%)	7 (24%)		4 (13%)	6 (43%)		2 (4.5%)	3 (21%)	
> 25%	9 (100%)	22 (76%)		27 (87%)	8 (57%)		42 (95%)	11 (79%)	
**TILs**			0.322			0.659			0.06
≤ 30%	6 (67%)	25 (86%)		27 (87%)	11 (79%)		22 (50%)	11 (79%)	
> 30 %	3 (33%)	4 (14%)		4 (13%)	3 (21%)		22 (50%)	3 (21%)	
**US Cortical Thickness**			0.130			0.053			0.102
< 7 mm	7 (78%)	13 (45%)		23 (74%)	6 (43%)		33 (75%)	7 (50%)	
≥ 7 mm	2 (22%)	16 (55%)		8 (26%)	8 (57%)		11 (25%)	7 (50%)	
**Depth Localization**			0.426			0.424			0.251
Posterior third	4 (44%)	8 (28%)		12 (39%)	4 (29%)		29 (66%)	6 (43%)	
Middle third	5 (56%)	16 (55%)		17 (55%)	7 (50%)		12 (27%)	6 (43%)	
Anterior third	0 (0%)	5 (17%)		2 (6.5%)	3 (21%)		3 (6.8%)	2 (14%)	
**Breast Composition**			0.422			0.265			0.27
A	1 (11%)	0 (0%)		1 (3.2%)	1 (7.1%)		2 (4.5%)	2 (14%)	
B	4 (44%)	14 (48%)		16 (52%)	11 (79%)		18 (41%)	7 (50%)	

C	3 (33%)	12 (41%)		8 (26%)	1 (7.1%)		12 (27%)	4 (29%)	
D	1 (11%)	3 (10%)		6 (19%)	1 (7.1%)		12 (27%)	1 (7.1%)	
**Background Parenchymal Enhancement**			>0.999			0.648			>0.999
Minimal/Mild	8 (89%)	23 (79%)		26 (84%)	13 (93%)		35 (80%)	12 (86%)	
Moderate/Marked	1 (11%)	6 (21%)		5 (16%)	1 (7.1%)		9 (20%)	2 (14%)	
**Margins**			0.128			0.92			0.737
Circumscribed / Irregular	6 (67%)	10 (34%)		16 (52%)	7 (50%)		32 (73%)	9 (64%)	
Spiculated	3 (33%)	19 (66%)		15 (48%)	7 (50%)		12 (27%)	5 (36%)	
**Shape**			>0.999			0.178			>0.999
Irregular	9 (100%)	27 (93%)		20 (65%)	12 (86%)		29 (66%)	10 (71%)	
Oval / Round	0 (0%)	2 (6.9%)		11 (35%)	2 (14%)		15 (34%)	4 (29%)	
**Intratumoral high SI on T2**			0.245			0.698			0.113
Present	5 (56%)	9 (31%)		7 (23%)	2 (14%)		17 (39%)	2 (14%)	
Absent	4 (44%)	20 (69%)		24 (77%)	12 (86%)		27 (61%)	12 (86%)	
**Peritumoral Edema**			>0.999			>0.999			0.052
Present	7 (78%)	20 (69%)		22 (71%)	10 (71%)		38 (86%)	8 (57%)	
Absent	2 (22%)	9 (31%)		9 (29%)	4 (29%)		6 (14%)	6 (43%)	
**Prepectoral Edema**			0.438			0.492			0.431
Present	2 (22%)	12 (41%)		11 (35%)	3 (21%)		21 (48%)	5 (36%)	
Absent	7 (78%)	17 (59%)		20 (65%)	11 (79%)		23 (52%)	9 (64%)	

**Subcutaneous Edema**			>0.999			>0.999			0.433
Present	2 (22%)	7 (24%)		4 (13%)	2 (14%)		7 (16%)	4 (29%)	
Absent	7 (78%)	22 (76%)		27 (87%)	12 (86%)		37 (84%)	10 (71%)	
**BES**			0.262			0.885			0.153
1	2 (22%)	8 (28%)		8 (26%)	4 (29%)		6 (14%)	5 (36%)	
2	5 (56%)	7 (24%)		11 (35%)	6 (43%)		15 (34%)	3 (21%)	
3	0 (0%)	7 (24%)		8 (26%)	2 (14%)		16 (36%)	2 (14%)	
4	2 (22%)	7 (24%)		4 (13%)	2 (14%)		7 (16%)	4 (29%)	
**Multifocality**			0.476			0.428			>0.999
Present	3 (33%)	14 (48%)		15 (48%)	5 (36%)		9 (20%)	3 (21%)	
Absent	6 (67%)	15 (52%)		16 (52%)	9 (64%)		35 (80%)	11 (79%)	
**Associated non-mass Enhancement**			0.254			>0.999			**0.009**
Absent	7 (78%)	15 (52%)		21 (68%)	10 (71%)		40 (91%)	8 (57%)	
Present	2 (22%)	14 (48%)		10 (32%)	4 (29%)		4 (9.1%)	6 (43%)	
**Internal Enhancement Type**			0.089			0.296			0.507
Homogeneous	0 (0%)	8 (28%)		16 (52%)	4 (29%)		10 (23%)	5 (36%)	
Heterogeneous	8 (89%)	14 (48%)		12 (39%)	7 (50%)		18 (41%)	6 (43%)	
Rim Enhancement	1 (11%)	7 (24%)		3 (9.7%)	3 (21%)		16 (36%)	3 (21%)	
**Delayed Phase Enhancement**			0.818			0.611			>0.999
Persistent	0 (0%)	2 (7%)		1 (3%)	1 (7%)		2 (5%)	1 (7%)	

Plateau	2 (22%)	10 (34%)		8 (26%)	2 (14%)		3 (7%)	1 (7%)	
Wash-out	7 (78%)	17 (59%)		22 (71%)	11 (79%)		39 (88%)	12 (86%)	
**Index Lesion MR Size**	25 (24, 34)	30 (24, 41)	0.667	28 (22, 38)	24 (20, 30)	0.173	25 (21, 42)	29 (21, 35)	0.993
**Maximal MR Size**	34 (25, 45)	40 (30, 73)	0.44	36 (25, 54)	33 (24, 60)	0.778	28 (21, 46)	39 (29, 54)	0.164
Median (IQR); n (%)									
Wilcoxon rank sum test; Fisher's exact test									
Wilcoxon rank sum test; Fisher's exact test; Pearson's Chi-squared test									

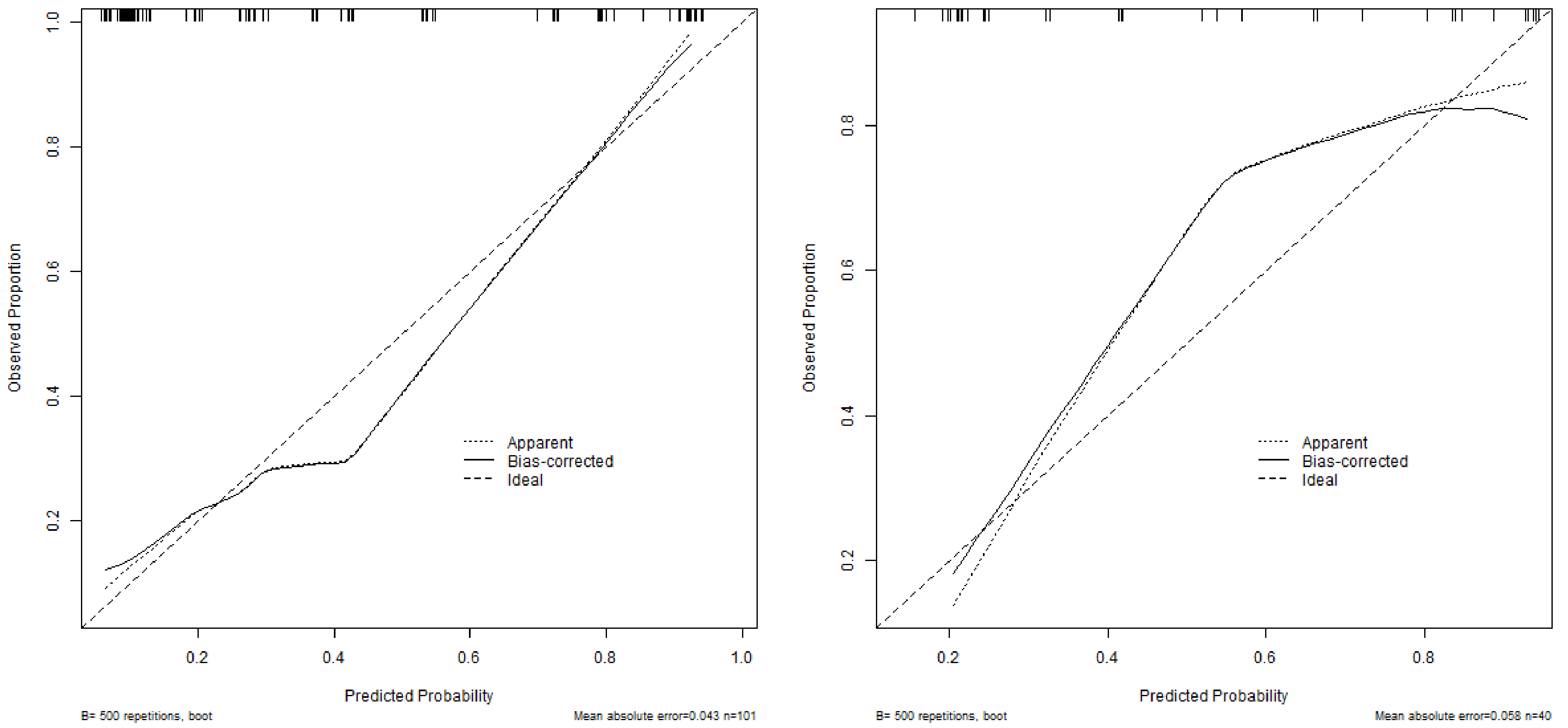

Figure S1: The calibration curves represent the relationship between the predicted lymph node status and the true lymph node status in the training (A) and the test sets (B). The x-axis represents the probability of positive LN status predicted by the model, and the y-axis represents the actual LN status. The dashed line at a 45° angle represents perfect calibration. The dotted line represents the predictive power of the model. Bootstrap overfitting–corrected calibration curve is shown with plain-line. The closer the calibration line is to the dashed line, the better the model's prediction.

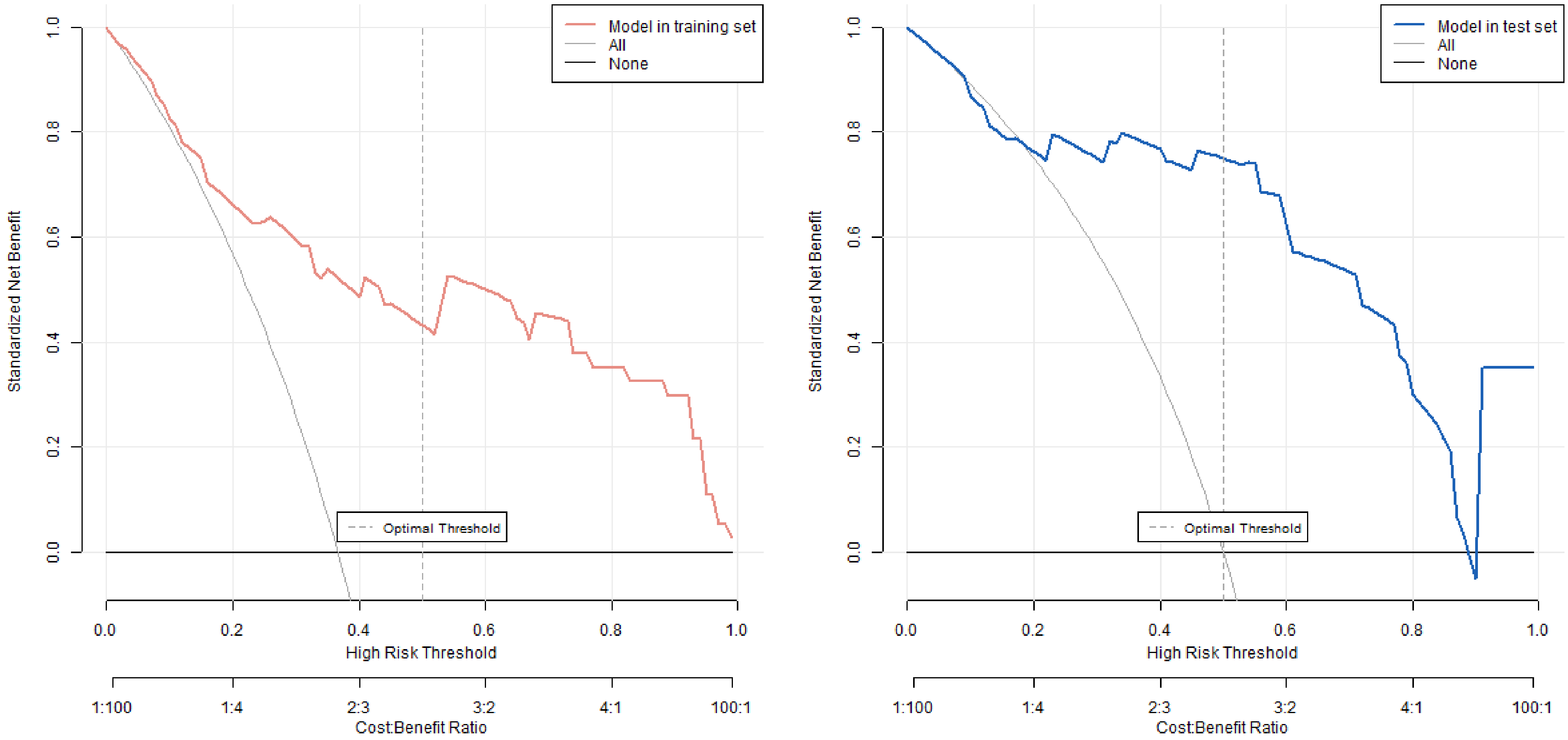

Figure S2: Decision curves of the multivariate model in the training (A) and test (B) sets. Decision curve analysis indicates a net benefit using the model in both sets for predicting axillary residual disease, above a threshold probability of 20% in the training test, and between 20% and 82% in the test set.
